# Supplementary material for: Active site alanine mutations convert deubiquitinases into high‐affinity ubiquitin‐binding proteins
Source: EMBO Rep. 2018 Aug 27;19(10):e45680. doi: 10.15252/embr.201745680 (PMC6172466; doi:10.15252/embr.201745680)
Supplement: Supplementary file 1 — Expanded View Figures PDF [file EMBR-19-e45680-s001.pdf]

Expanded View Figures

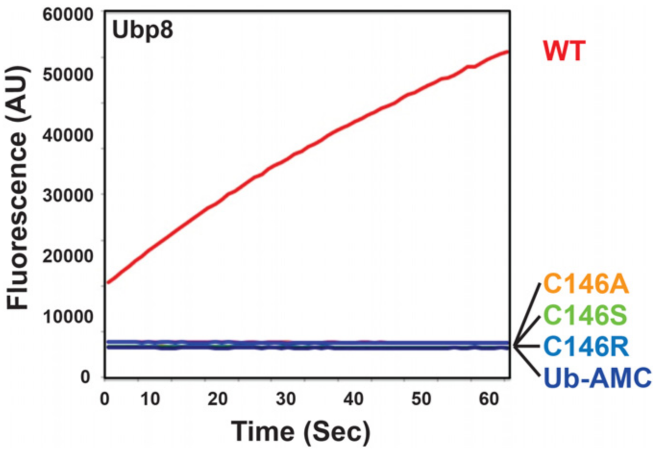

Figure EV1. Catalytic activity of DUBm-Ubp8 mutants.

Progress curve of Ub-AMC cleavage by 125 nM DUBm-Ubp8^{WT} or mutants. The experiment as shown was performed once. The C146A and C146S mutants have been shown to lack any cleavage activity in at least two experiments each performed by other assay methods.

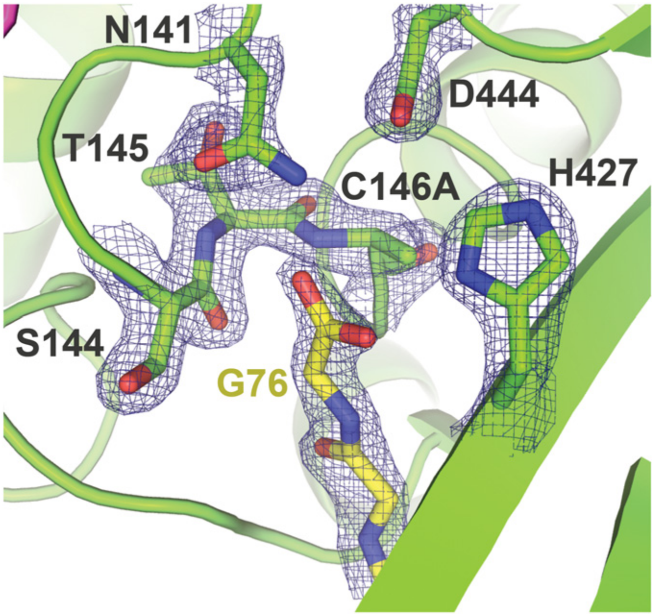

Figure EV2. Active site of DUBm-Ubp8^{C146A} bound to monoubiquitin.

Density is rendered from the $2F_o–F_c$ map and contoured at 2σ.

© 2018 The Authors

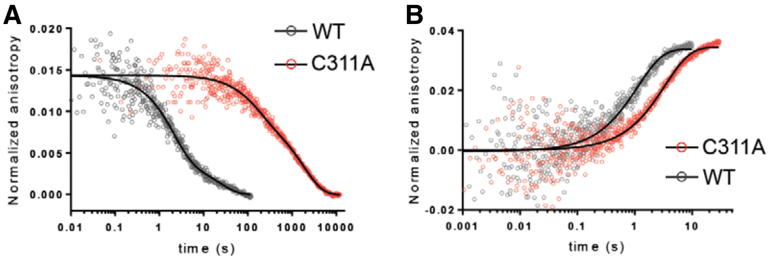

	K_{slow} (s^{-1})	K_{fast} (s^{-1})
**USP4 wt**	0.045 ± 0.01	0.49 ± 0.04
**USP4 CA**	$5.5x10^{-4}$ ± $3.5x10^{-5}$	$6.3x10^{-3}$ ± $6.0x10^{-4}$

	K_{on} (μM^{-1} s^{-1})
**USP4 wt**	0.29 ± 0.01
**USP4 CA**	0.88 ± 0.05

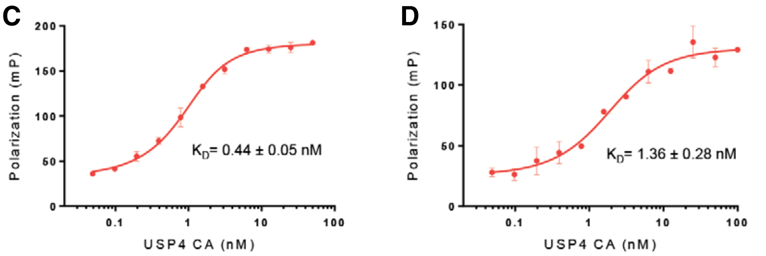

Figure EV3. Binding kinetics of USP4 WT and C311A to ubiquitin substrates.

A Dissociation kinetics of USP4 WT and C311A binding to TAMRA-monoubiquitin, measured by stopped-flow fluorescence polarization.

B Association kinetics for USP4 WT and C311A binding to TAMRA-monoubiquitin. Measured by stopped-flow fluorescence polarization.

C Equilibrium binding of USP4 C311A to TAMRA-ubiquitin conjugated to lysine.

D Equilibrium binding of USP4 C311A to TAMRA-ubiquitin conjugated to an 18-mer peptide derived from SMAD4.

Data information: The error bars in panels (C) and (D) are s.d. calculated on two measurements per point.

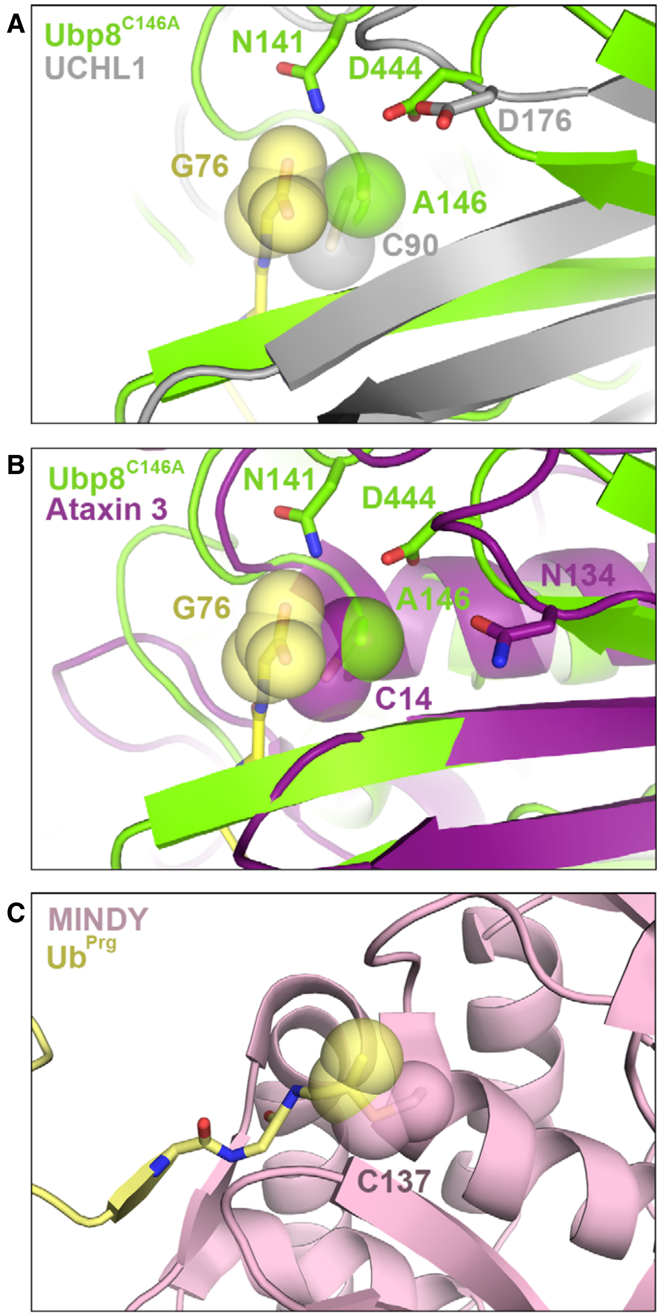

Figure EV4. Alignment of the active site of Ubp8^{C146A}+monoUb to other cysteine protease DUBs.

A, B Alignment of Ubp8^{C146A}+monoUb active site to (A) ubiquitin-bound UCHL1 (PDB ID 3KW5) or (B) ubiquitin-bound Ataxin 3 (PDB ID 3O65).

C The active site of ubiquitin propargyl-bound MINDY (PDB ID 5JQS) shown alone, as it does not align with Ubp8^{C146A} due to a lack of structural conservation.

© 2018 The Authors
